# *CCNI2* plays a promoting role in the progression of colorectal cancer

**DOI:** 10.1002/cam4.3504

**Published:** 2021-02-23

**Authors:** Dong‐Ming Lai, Jiang‐Jiang Bi, Yong‐Hui Chen, Yu‐Di Wu, Qing‐Wen Huang, Hai‐Jie Li, Sheng Zhang, Zheng Fu, Yi‐Xin Tong

**Affiliations:** ^1^ Department of Gastrointestinal Surgery Sun Yat‐sen memorial hospital affiliated Sen Yat‐sen University Guangzhou China; ^2^ Department of Anesthesiology Tongji Hospital of Tongji Medical College of Huazhong University of Science and Technology Wuhan Hubei China; ^3^ Department of GI Surgery Tongji Hospital of Tongji Medical College of Huazhong University of Science and Technology Wuhan Hubei China; ^4^ Department of Pharmacology University of Virginia Charlottesville VA USA

**Keywords:** apoptosis, *CCNI2*, colorectal cancer, proliferation

## Abstract

Colorectal cancer (CRC) is one of the most common malignancies and most of the patients diagnosed with advanced CRC have unsatisfactory treatment effect and poor prognosis. The purpose of this study was to investigate the effect of *CCNI2* on the development of CRC. In this sutdy, immunohistochemical staining was used to detect *CCNI2* expression levels in clinical samples, meanwhile, the Kaplan‐Meier survival analysis was conducted. Celigo cell counting assay was used for screening shCCNI2s. QPCR and WB were performed to verify knockdown efficiency of *CCNI2*. Cell proliferation, colony formation, cell cycle, apoptosis, and mechanism investigation of *CCNI2* knockdown were investigated by MTT assay, colony formation assay, fluorescence‐activated cell sorting, and human apoptosis antibody array, respectively. Otherwise, the mouse model of *CCNI2* knockdown was also constructed. The results of immunohistochemical staining and qPCR indicated that *CCNI2* had a high expression level in the CRC tissues and cell lines. Kaplan‐Meier survival analysis manifested that the high expression of *CCNI2* suggested poor prognosis. The expression of *CCNI2* was significantly reduced by *CCNI2*‐siRNAs, and the downregulated expression level of *CCNI2* inhibited CRC cell proliferation and colony formation, arrested cell cycle in G2 phase, as well as promoted cell apoptosis. The various indexes of solid tumor in mice models indicated that *CCNI2* knockdown could suppress the growth of CRC tumor. Based on the comprehensive analysis of the above results, *CCNI2* was contributed to the progression of CRC and could serve as a prognostic marker for CRC.

## Graphical abstract

The influence of CCNI2 knockdown on CRC in vivo. Fluorescence images showed the growth of tumors in mice models

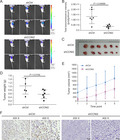

## 1 INTRODUCTION

Colorectal cancer (CRC) is the fourth leading cause of cancer‐related death worldwide, and the third most common malignant tumor.^1^ According to statistics, the number of global CRC deaths in 2018 is about 881,000, which is expected to increase to 1.1 million by 2030.^2^ In China, there are 1.8 million new cases in 2018, ranking third in the incidence rate, accounting for one‐tenth of all cancer cases, and it is still rising steadily.^3^, ^4^ CRC is characterized by heterogeneity of changes in the genome, epigenome, and transcriptome.^5^ Plenty of evidence suggested that genetic mutations, epigenetic modifications, chronic inflammation, diet, lifestyle, and intestinal flora are critical risk factors for CRC, with genetic factors playing a vital role in both familial and sporadic etiology.^6^, ^7^ Among them, up to 35% of variation in the risk of CRC is attributable to genetic factors.^8^ Nakanishi Y et al. proposed that CRC was mainly developed from precursor lesions of traditional or alternative pathways. The traditional pathway was associated with adenomatous polyposis coli (APC) tumor suppressor gene inactivation, and another pathway was associated with activation of the ERK cascade, occasionally due to activation of *KRAS* or *BRAF* mutations.^9^ Previous studies has demonstrated that mutant *KRAS* synergizes could promote CRC cells growth and self‐renewal, while *SLC25A22* promoted proliferation of CRC cells with *KRAS* mutation.^10^

Approximate 90% of patients diagnosed in the early stages of CRC are curable through surgery.^7^ However, due to the high heterogeneity of CRC and frequent mutations, patients with CRC are usually diagnosed at an advanced stage, thus, accompanied with poor prognosis.^7^, ^11^ More than 90% of patients with CRC metastasis die within 5 years of diagnosis.^12^ Early screening can reduce the incidence and mortality of CRC by removing precancerous adenomatous polyps and detecting cancer at an earlier, more treatable stage, which requires to clarify the specific molecular mechanism of the occurrence and development of CRC.^13^ Although much effort has been made over the past several decades, the underlying molecular mechanism of the occurrence and development of CRC remains unclear.^7^

Cyclins are a regulatory subunit of cyclin‐dependent kinases (CDK), and the members of cyclin protein family are characterized by a conserved region through which they bind to cyclin‐dependent kinases, the cyclin box, to form a complex that control cell cycle progression.^14^, ^15^ Originally cloned from the human forebrain cortex, Cyclin I (*CCNI*) belongs to a new subgroup of the cyclin family together with Cyclin G1 and G2, and has 30% amino acid homology with Cyclin G1 and G2.^16^, ^17^, ^18^ Recent studies have shown that the expression level of *CCNI* was related to the proliferation and angiogenesis of human cancer. Downregulation of *CCNI* can inhibit cell proliferation by arresting the cell cycle at S and G2/M phase.^17^ Sian V. Griffin et al. also reported that *CCNI* regulated podocyte apoptosis both *in vitro* and a model of glomerular diseases.^15^

Cyclin I‐like (*CCNI2*) is considered to be a homolog of *CCNI*, although the overall similarity of the two proteins is relatively low except for the cyclin box. It is found that *CCNI* is mainly located in the nuclei while *CCNI2* is mainly in the cytoplasm and plasma membrane.^19^ By analyzing all cyclin genes expression patterns in human islets, Jalal Taneera et al. found that all cyclin genes expression levels were higher than background control values, with *CCNI* expression level being the highest and *CCNI2* expression level being relatively low.^20^ It has also been reported that cell cycle progression and proliferation are inhibited after knockdown of *CCNI2*. However, the physiological function of *CCNI2* in cancer is still unclear and its role in CRC is rarely reported.^19^ In this study, we found *CCNI2* had a high expression level in CRC. *In vitro* and *in vivo*, we investigated the effects of *CCNI2* on CRC cells proliferation, cell cycle, and apoptosis, and initially explored the molecular mechanism of *CCNI2* in CRC.

## 2 MATERIALS AND METHODS

### 2.1 Tissues samples

Paraffin‐embedded CRC tissues microarray (HColA180Su15), including the pathological characteristics of tissues samples, was purchased from Shanghai Outdo Biotech Co., Ltd. (Shanghai, China). All of the donors signed the informed consent form and experiments were approved ethical approval by the ethics committee of Huazhong University of science and technology.

### 2.2 Cell culture

Human normal colorectal mucosal cells FHC (CRL‐1831) were purchased from BeNa Technology (Hangzhou, Zhejiang, China), and human CRC cell lines CACO2 (TCHu146), RKO (TCHu116), SW480 (TCHu172), and HCT 116 (TCHu 99) were purchased from Cell Bank, Chinese Academy of Science (Shanghai China). FHC, CACO2, and HCT 116 cells were maintained in 90% RPMI 1640 (corning, Corning, NT, USA.) with 10% of fetal bovine serum (FBS, Invitrogen, Carlsbad, California, USA). RKO cells were maintained in 90% DMEM with 10% FBS. SW480 cells were maintained in 90% L‐15 with 10% FBS. All cells were cultured the incubator (MCO‐175, SANYO Electric Co., Ltd, Osaka, Japan) with 5% CO_2_ at 37°C.

### 2.3 Immunohistochemical staining

The tumor tissues were fixed in 10% of neutral formalin for 12 h, and then, dehydrated, transparent, and embedded, and made into paraffin sections with a thickness of 5 μm for immunohistochemical staining. After being dewaxed, rehydrated, and blocked, slides were incubated with CCNI2 antibody (1:100, ab97767, Abcam, Cambridge, MA, USA) or Ki67 antibody (1:200, Ab16667, Abcam, Cambridge, MA, USA) at 4°C overnight, then, washed with phosphate‐buffered saline (PBS) for 3 times, and incubated with horseradish peroxidase (HRP) conjugated goat anti‐rabbit IgG polyclonal antibody (1:400, ab6721, Abcam, Cambridge, MA, USA) at room temperature for 30 min. 3,3′‐diaminobenzidine (DAB) was applied to stain tissue slides at room temperature for 5 min in the dark, and hematoxylin (Baso Diagnostics Inc., Zhuhai, China) was used to counterstain for 10‐15 s. Photomicroscope (Olympus IX73) was used to observe and capture images. At least 10 representative fields (× 200 magnification) were selected to count the number of staining positive cells and determine the staining intensity.

### 2.4 Target gene Knockdown cell model

Using *CCNI2* gene (NM_001287253.1) as a template, RNA interference target sequences were designed: shCCNI2‐1, 5’ ‐ ATCTGCGACGCCTTCGAGGAA ‐ 3’; shCCNI2‐2, 5’ ‐ TACCTGCATTGCGCCACAATT ‐ 3’; shCCNI2‐3, 5’ ‐ CCTGGAAGGCGACCTGGACGA ‐ 3'. The RNA interference target sequence of negative control was designed: shCtrl, 5’ ‐ TTCTCCGAACGTGTCACGT ‐ 3'. Then, target *CCNI2* RNA interference sequences were cloned into BR‐V‐108 lentiviral vector (Shanghai Yibeirui Biomedical Science and Technology Co., Ltd) containing Age I/EcoR I enzyme cutting site, and the 293 T (635) cells were co‐transfected with BR‐V‐108, pHelper 1.0 (Shanghai Yibeirui Biomedical Technology Co., Ltd) and pHelper 2.0 (Shanghai Yibeirui Biomedical Technology Co., Ltd). After 48 h, cells were centrifuged at 4000 g for 10 min at 4°C, the supernatant containing the virus was harvested, concentrated, and purified, and then, stored in the virus preservation solution. Human CRC cell lines HCT 116 and RKO were trypsinized, resuspended, and then, seeded into 6‐well plate (2 × 10^5^ cells/well). A 400 μL lentiviral vectors (1 × 10^7^ TU/well) were added into the plate. After 72 h, fluorescence microscope (OLYMPUS, Tokyo, Japan) was used to observe the fluorescence of cells and evaluate infection efficiency. Maps of BR‐V‐108, pHelper 1.0, and pHelper 2.0 were provided in Figure S1.

### 2.5 QPCR

Total RNAs in CRC cells and tissues were extracted with Trizol Reagent (Invitrogen, Carlsbad, CA, USA). Then, 1 μL Oligo dT (0.5 μg/μL) and 2.0 μg total RNAs were reversed transcription to obtain cDNA by Promega M‐MLV Kit (Promega Corporation, Heidelberg, Germany). RNA levels were detected by Real‐Time PCR Detecting System (VII7, Applied Biosystems, Waltham, Mass, USA) and the relative quantitative analysis of RNA was calculated by the formula: 2^−∆∆CT^. The effective upper limit of CT value for RNA detection by real‐time PCR detection system was 35. GAPDH was used as an internal control. The forward primer and reverse primer sequences used in this experiment were as follows: *CCNI2*: 5’ ‐ CCAGGGAGTATGAATGAATGTT ‐ 3’ and 5’ ‐ TTGGGATAAGCCTGGGAAGTT ‐ 3’; *GAPDH*: 5’ ‐ TGACTTCAACAGCGACACCCA ‐ 3’ and 5’ ‐ CACCCTGTTGCTGTAGCCAAA ‐ 3’. PCR conditions were set as 95°C 30 s, 1 cycle; 95°C 5 s, 60°C 30 s, 40 cycle. This experiment was done in triplicate.

### 2.6 Western blot

After infected with *CCNI2* shRNA, HCT 116, and RKO cells were collected and lysed by Cell Lysis Buffer (9803S, Cell Signal Technology, Danvers, MA). BCA Protein Assay Kit (23225, HyClone‐Pierce, Logan, UT, USA) was used to measure protein concentration. The total cellular proteins (20 μg) were subjected by 10% SDS‐PAGE for western blot (WB) analysis, and transferred to polyvinylidene difluoride (PVDF, IPVH00010, Millipore Life Science, Boston, MA, USA) membranes by wet transfer. Membranes were blocked by TBST with 5% skim milk at 4°C for 1 h, and then incubated in primary antibodies (CCNI2 antibody: 1:1000, ab97767, Abcam, Cambridge, MA, USA; GAPDH antibody: 1:3000, AP0063, Bioworld, MN, USA) at 4°C overnight. After that, horseradish peroxidase (HRP)‐conjugated goat anti‐rabbit IgG polyclonal antibody (1:3000, A0208, Beyotime Biotechnology, Shanghai, China) as the secondary antibody was used to incubate the membranes for 2 h at room temperature. ECL‐Plus^TM^ Western blotting system kit from Amersham (RPN2232, Chicago, IL, USA) was used for color developing, and chemiluminescence imaging system (AI600, GE Healthcare Life Sciences, USA) was employed to take photos, and Image J (National Institutes of Health, Bethesda, Maryland, USA) was used for immunoblotting density analysis.

### 2.7 High content screening (HCS)‐Celigo cell counting assay

RKO cells, infected with lentivirus‐shCCNI2 s, were trypsinized, resuspended and counted 2000 cells that were seeded into 96‐well plate. Then the cells were cultured in an incubator with 5% CO_2_ at 37°C. After 24 h, Celigo (Nexcelom Bioscience, Lawrence, Massachusetts, USA) was used to scan the 96‐well plate at the same time for five consecutive days to obtain the scanning images, and Image J was used to count the cells in the scanning images.

### 2.8 MTT assay

The HCT 116 and RKO cells infected with *CCNI2* shRNA or control shRNA were resuspended and transferred to the 96‐well plate (2000 cells/well). 20 μL MTT (5 mg/mL; Genview, Florida, USA) were added per well for 4 h, then, 100 μL dimethyl sulfoxide (DMSO; Shanghai, China) were added into each well for 5 min and finally, the absorbance of cell suspension was detected by microplate reader (Tecan infinite, Männedorf, Zürich, Switzerland) at the wavelength of 490 nm. This assay was done in triplicate.

### 2.9 Colony formation assay

Infected with *CCNI2* shRNA for 5 days, HCT 116 and RKO cells were resuspended and seeded into 6‐well plate with 500 cells/well. Cultured continuously for 8 days, cells were immobilized with 1 mL 4% paraformaldehyde for 60 min, stained with 500 μL GIEMSA (Dingguo Biotechnology, Shanghai, China) for 15 min and photographed with a digital camera. A population of cell (> 50 cells) derived from a single cell was called a clone. The proliferative potential of individual cells was assessed by counting clone formation rate. The experiment was carried out at least three times under the same conditions.

### 2.10 Fluorescence activated cells sorting (FACS)

The cell apoptosis rate of HCT 116 and RKO cells infected with shRNA lentivirus was determined by fluorescence activated cells sorting. Cells were harvested when the cell fusion degree reached 80%, and washed with D‐Hanks (pH =7.2 ~ 7.4) precooled at 4°C. After centrifuged, cells were resuspended by 200 μL 1 × binding buffer and incubated with 10 μL Annexin V‐APC (88‐8007, eBioscience, California, USA) for 15 min in the dark. The apoptosis rate was assessed by FACScan (Guava easyCyte HT, Millipore, Schwalbach, Germany). This experiment was repeated at least three times.

The cell cycle distribution of HCT 116 and RKO cells after CCNI2 knockdown was detected by fluorescence activated cells sorting. After 5 days infected with shCCNI2‐lentivirus, HCT 116, and RKO cells were treated with trypsin and collected by centrifugation. Then cells were fixed in 70% ethanol for 1 h after washing with precooled PBS and then centrifuged. Afterward, 1.0 mL staining solution was added to resuspended cells at a proportion 40 × PI (2 mg/mL, P4170, Sigma):100 × RNase (10 mg/mL, 2158‐1, TakaPa):1 × PBS =25:10:1000. In the end, the cell cycle distribution was measured by FACScan (Guava easyCyte HT, Millipore). This assay was repeated at least three times.

### 2.11 Human apoptosis antibody array

Human apoptosis signaling pathway‐related genes were detected by Human Apoptosis Antibody Array (ab134001, Abcam, Cambridge, MA, USA) in RKO cells infected with shRNA lentivirus. Each membrane printed side up was placed into the 8‐well tray, blocked with 2 mL 1 × Blocking Buffer for 30 min at room temperature, and incubated in 1.2 mL cell lysis overnight at 4°C. After that, membranes were incubated in 1 mL 1 × Biotin‐Conjugated Anti‐Cytokines overnight at 4°C. Added 1.5 mL 1 × Streptavidin‐HRP to incubate for 2 h at room temperature after washed. Signals were detected using enhanced chemiluminescence (ECL). Chemiluminescence imaging system (AI600, GE Healthcare Life Sciences, USA) was performed to take photos, and Image J (National Institutes of Health, Bethesda, Maryland, USA) was employed for immunoblotting density analysis. Each sample was done in duplicate.

### 2.12 Tumor‐bearing animal model

BALB/c nude mice (4‐week old, male) were purchased from Shanghai SLAC Laboratory Animal CO. LTD, which were raised in stainless steel cages with room temperature of 24°C and relative humidity of 70%. Mice could drink filtered tap water and commercial feed at will. The animal laboratory is cleaned twice one day and sterilized with ultraviolet light for 1 h every week. For the study, mice were divided into two group: NC group and shCCNI2 group (six mice in each group). RKO cells in the logarithmic growth phase, infected with shRNA lentivirus, were resuspended and 200 μL cell suspension (4 × 10^6^ cells) was injected subcutaneously into the right forelimb of the mice. 13 days later, observe the tumor formation. The volume of tumors was measured by Vernier caliper and the weight of mice was measured by counter balance every 3 days. On the 28^th^ day after subcutaneous injection, 15 mg/mL D‐Luciferin (10 μL/g) were injected into the abdominal cavity of mice, and 15 min later, 0.7% sodium pentobarbital (10 μL/g) were used to anesthetize mice by intraperitoneal injection. Mice were placed under a live imager (LB 983, BERTHOLD TECHNOLOGIES GmbH & Co. KG, Bad Wildbad, Baden‐Wurttemberg, Germany) for imaging and observing bioluminescence. Then, all mice were sacrificed and the tumors were removed and frozen. The volume and weight of tumors were measured. Besides, Ki‐67 expression levels were determined by immunohistochemical staining to evaluate the tumor proliferation index. All of the animal experiments were approved by the Institutional Animal Ethics Committee.

### 2.13 Statistical analysis

All experiments on cell levels in this study were performed in triplicate. All date in this study were analyzed *via* GraphPad Prism 6 (San Diego, CA, USA) and results were showed as mean ±standard deviation (SD). Sign test was employed for the statistical analysis of the CCNI2 levels in CRC tissues and paracarcinoma tissues. Mann‐Whitney U analysis and Spearman rank correlation test were applied for the correlation between *CCNI2* expression and tumor characteristics of CRC patients. LogRank test was used to assess the statistical significance of the association between *CCNI2* levels and overall survival of CRC patients. Unpaired *t*‐test (two‐tailed) was used for statistical analysis after F test. *p* < 0.05 was considered statistically significant.

## 3 RESULTS

### 3.1 *CCNI2* was significantly upregulated in CRC tissues and cell lines

**Figure 1:**
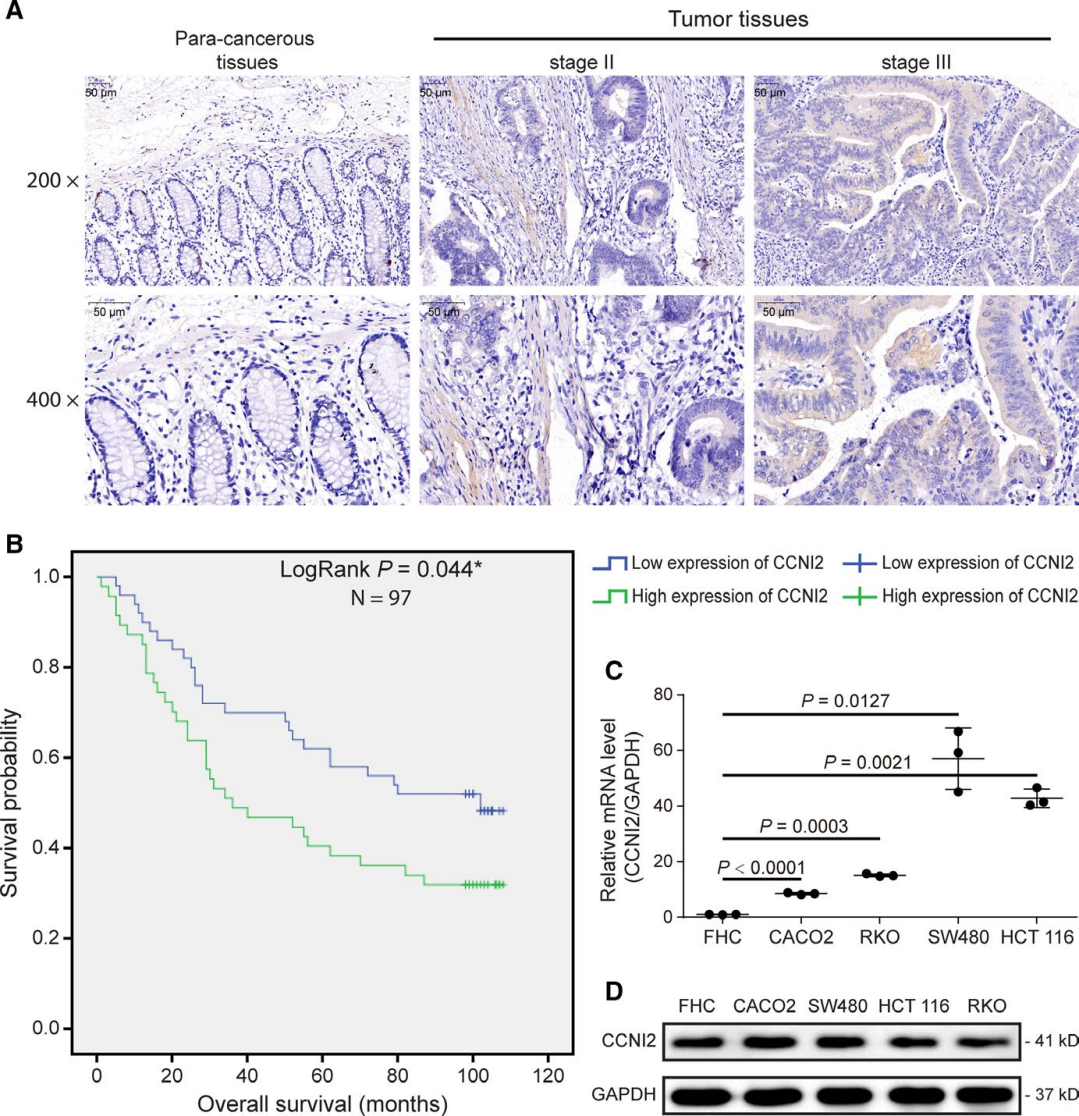
The expression of *CCNI2* in colorectal cancer (CRC) tissues and cell lines. (A) The expression levels of *CCNI2* in clinical tissue samples were detected by immunohistochemical staining and the results demonstrated that CCNI2 was upregulated in CRC tissues compared with paracancerous tissues. The magnification was 200 times (scale bar =50 μm) and 400 times (scale bar =50 μm). (B) Kaplan‐Meier survival analysis indicated that the expression of *CCNI2* was significantly correlated with the overall survival of CRC patients: the high expression level of *CCNI2* suggested the poor prognosis of CRC patients was decreased. Statistical analysis was performed by LogRank test. (C) The relative expression of *CCNI2* in human normal colorectal mucosal cells (FHC) and several CRC cell lines (CACO2, RKO, SW480, and HCT 116) were detected by qPCR. ∆Ct =the Ct of *CCNI2* ‐ the Ct of GAPDH. Statistical analysis was performed by unpaired *t*‐test (two‐tailed). (D) The protein levels of CCNI2 in human normal colorectal mucosal cells (FHC) and several CRC cell lines (CACO2, HCT 116, SW480, and RKO) were detected by WB.

The immunohistochemical staining indicated that CCNI2 was highly expressed in CRC tissues compared to paracarcinoma tissues (Figure 1A, Table 1). Meanwhile, statistical analysis of the correlation between *CCNI2* expression and tumor characteristics in patients with CRC and Spearman rank correlation analysis also showed that there was a positive correlation between *CCNI2* expression and the pathological grading of CRC (Tables 2, 3). Furthermore, Kaplan‐Meier survival analysis revealed the association between high *CCNI2* expression and poor prognosis of CRC patients (Figure 1B). All of these results suggested that *CCNI2* might involve in the development and progression of CRC and had the potential to act as a prognosis indicator for CRC. Furthermore, the results of qPCR and WB assays revealed that compared with the human normal colorectal mucosal cells FHC, the mRNA and protein levels of *CCNI2* were dramatically increased in CRC cell lines (CACO2, RKO, SW480, and HCT 116), showed in Figure 1C‐D and Figure S2A‐S2B, which was consistent with the results of detection of CCNI2 expression in the tissue microarray of clinical samples. RKO and HCT 116 cell lines were chosen for subsequent analysis.

**Table 1:** Expression patterns in colorectal cancer tissues and paracarcinoma tissues revealed in immunohistochemistry analysis.

CCNI2 expression	Tumor tissue		Paracarcinoma tissue		*p* value
	Cases	Percentage	Cases	Percentage	
Low	50	51.5%	59	100%	0.049*
High	47	48.5%	0	‐	

The statistical analysis was performed by Sign test.

**Table 2:** Relationship between *CCNI2* expression and tumor characteristics of patients with colorectal cancer

Features	No. of patients	CCNI2 expression		*p* value	Statistics
		low	high		
All patients	97	50	47		
Age (years)				0.350	Mann‐Whitney U
≤71	47	26	21		
＞71	44	20	24		
Gender				0.512	Mann‐Whitney U
Male	53	26	27		
Female	43	24	19		
Lymph node positive				0.496	Mann‐Whitney U
≤0	47	27	20		
＞0	38	19	19		
Tumor size				0.157	Mann‐Whitney U
≤5 cm	47	21	26		
＞5 cm	49	29	20		
Grade				0.012*	Mann‐Whitney U
II	50	32	18		
III	47	18	29		
Stage				0.647	Mann‐Whitney U
1	5	3	2		
2	52	27	25		
3	36	20	16		
4	3	0	3		
T Infiltrate				0.789	Mann‐Whitney U
T1	1	1	0		
T2	5	3	2		
T3	74	37	37		
T4	13	7	6		
lymphatic metastasis（N）				0.745	Mann‐Whitney U
N0	57	30	27		
N1	27	14	13		
N2	11	5	6		
Lymph nodes				0.681	Mann‐Whitney U
＜7	42	22	20		
≥ 7	44	25	19		

Mann‐Whitney U analysis were applied for the correlation between *CCNI2* expression and tumor characteristics of CRC patients.

**Table 3:** Relationship between *CCNI2* expression and tumor characteristics of patients with colorectal cancer

		CCNI2
Grade	Correlation coefficient	0.257
	Significance (two‐tailed)	0.011*
	N	97

Spearman rank correlation test was applied for the relationship between *CCNI2* expression and tumor characteristics of CRC patients.

### 3.2 The *CCNI2* knockdown crc cell model was successfully constructed *in vitro*

There were three shRNAs of *CCNI2* used in this study and shCCNI2 s were infected into CRC cell lines HCT 116 and RKO with lentivirus infection technique. The detection of the fluorescence of green fluorescent protein (GFP) tagged on lentivirus vector verified that the infection efficiencies were >80% for both cell lines (Figure S3A). The qPCR results revealed that the relative *CCNI2* expression was lowest in shCCNI2‐2 group, in which the knockdown efficiency reached 90.1% (Figure S3B). Moreover, the results of Celigo cell counting assay showed that, compared with other shCCNI2 s, the proliferation rate of RKO cells infected with shCCNI2‐2 slowed down significantly, indicating that shCCNI2‐2 had the most significant inhibitory effect on the proliferation of RKO cells (Figure S3C‐D). Combined with the expression of *CCNI2* in RKO cells after shCCNI2 s infection, shCCNI2‐2 was used for the following study. Besides, the qPCR and WB results revealed that *CCNI2* mRNA and protein levels were significantly decreased in shCCNI2 group (Figure S3E‐F and Figure S4A‐D). Collectively, the *CCNI2* knockdown CRC cell models were successfully constructed *in vitro*.

### 3.3 Knockdown *CCNI2* suppressed crc cell proliferation, arrested cell cycle, and promoted apoptosis

**Figure 2:**
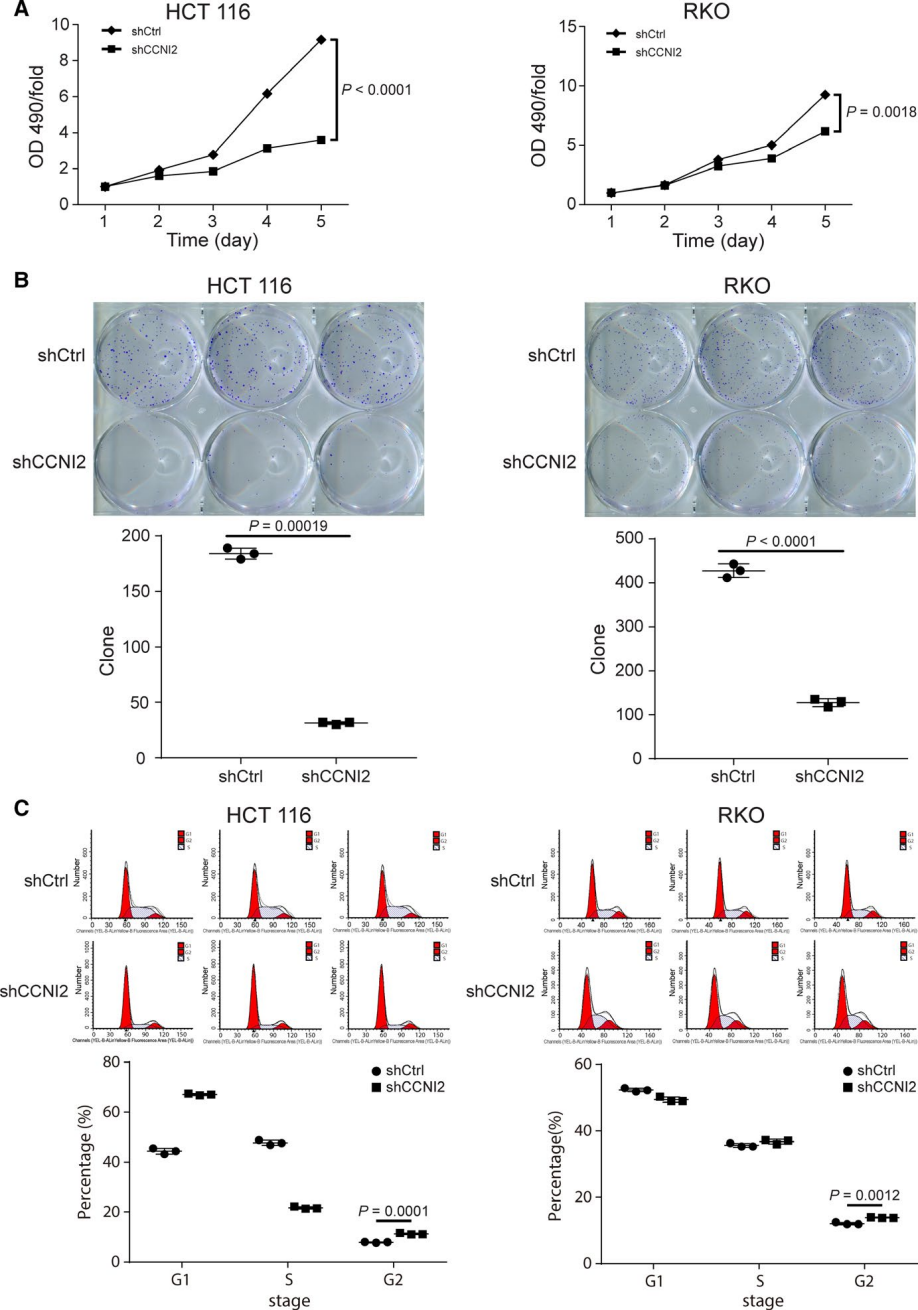
The effects of *CCNI2* knockdown on CRC cells proliferation and cell cycle. (A) Cell proliferation was measured by MTT. The fold change was absorbance for five consecutive days compared to absorbance at day 1. The last detecting results were used for statistical analysis to calculate the *P* value. Statistical analysis was performed by unpaired *t*‐test (two‐tailed). (B) Colony formation in HCT and RKO cells was significantly inhibited after *CCNI2* knockdown, as assessed by colony formation assay. Statistical analysis was performed by unpaired *t*‐test (two‐tailed). (C) Cell cycle distribution of HCT 116 and RKO cells was determined by fluorescence‐activated cells sorting. The results showed that the radio of G2 phase in cell cycle increased significantly after *CCNI2* knockdown. Statistical analysis was performed by unpaired *t*‐test (two‐tailed). ShCtrl: cells infected with scrambled control shRNA; shCCNI2: cell infected with *CCNI2*‐shRNA

**Figure 3:**
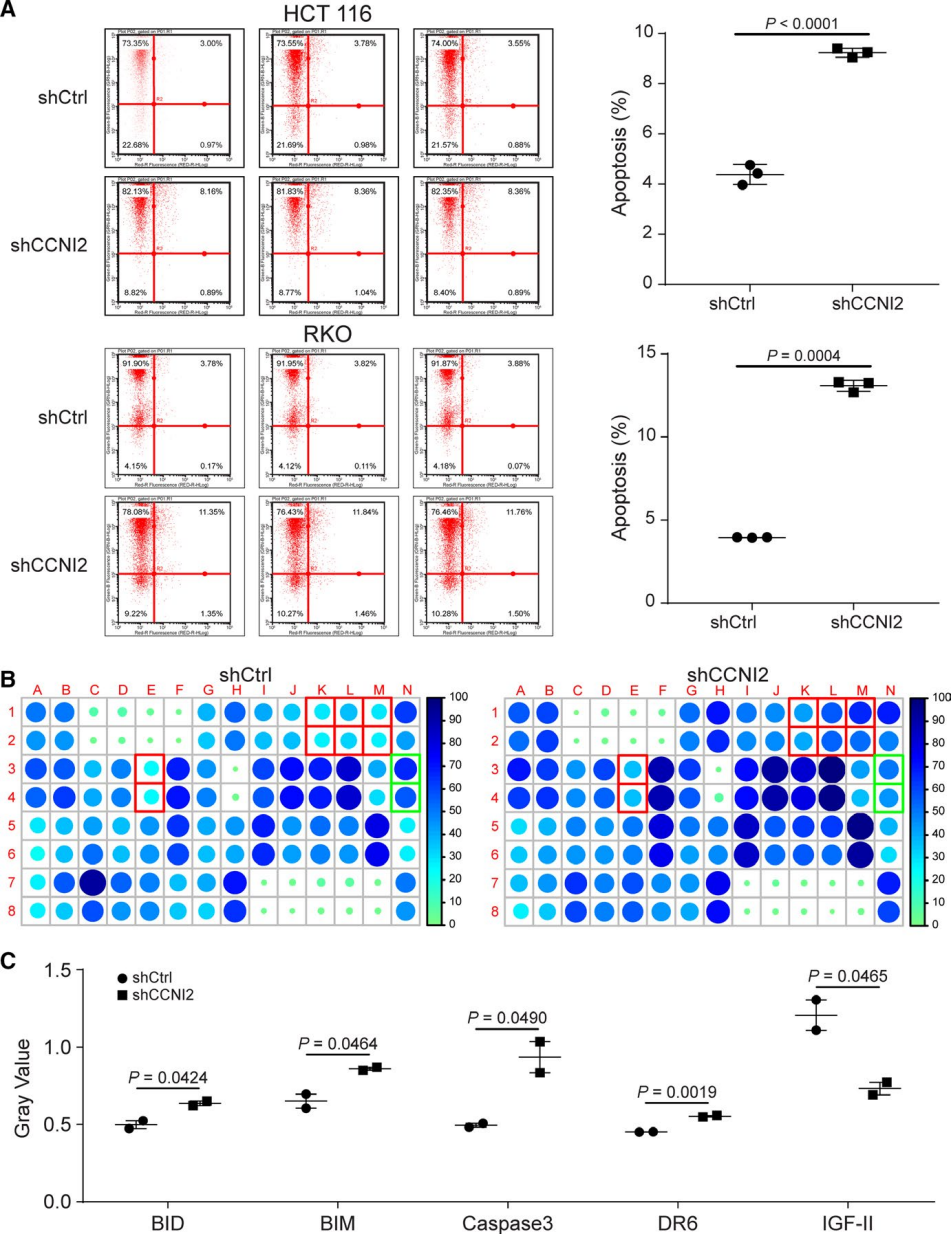
Mechanism study of *CCNI2* knockdown in CRC cells. (A) *CCNI2* knockdown dramatically increased CRC cells apoptosis rate. Statistical analysis was performed by unpaired *t*‐test (two‐tailed). (B) Intracellular signaling array after shCCNI2 infection. Red: expression level of protein increased; green: expression level of protein decreased. (C) *CCNI2* knockdown significantly increased the expression levels of BID, BIM, Caspase3, and DR6, and decreased the expression level of IGF‐II. Statistical analysis was performed by unpaired *t*‐test (two‐tailed). ShCtrl: cells infected with scrambled control shRNA; shCCNI2: cells infected with *CCNI2*‐shRNA.

Subsequently, the influences of *CCNI2* knockdown on CRC cell function were detected. MTT assay was performed to evaluate the effect of *CCNI2* knockdown on cell proliferation. The results indicated that, compared with the negative control (shCtrl group), knockdown of *CCNI2* significantly suppressed cell proliferation of HCT 116 and RKO cells (Figure 2A). Colony formation assay also showed that the colony formation ability of CRC cells was inhibited by *CCNI2* knockdown (Figure 2B). Furthermore, results of fluorescence activated cells sorting demonstrated that *CCNI2* silencing not only blocked cell cycle in G2 phase (Figure 2C), but also increased cell apoptosis rate, suggesting that knockdown of *CCNI2* could induce CRC cell apoptosis (Figure 3A). The FACS gating strategy of the representative plot was provided in Figure S5A‐B.

### 3.4 Mechanism study *CCNI2* knockdown in crc cells was explored by antibody array

For exploring the potential regulation mechanism of *CCNI2* on CRC, human apoptosis antibody array was applied to identify the differential expressed proteins in RKO cells between shCCNI2 and shCtrl groups. The distribution of apoptosis markers in the antibody array was displayed in Figure 3B. Accordingly, the upregulated expression levels of BID, BIM, Caspase3, and DR6, and the downregulated expression of IGF‐II were observed (Figure 3B‐C and Figure S6A‐B). Therefore, the reasonable conjecture that *CCNI2* affected CRC cell through regulating these proteins could be supposed.

### 3.5 Effects of *CCNI2* knockdown on CRC were investigated *in vivo*

**Figure 4:**
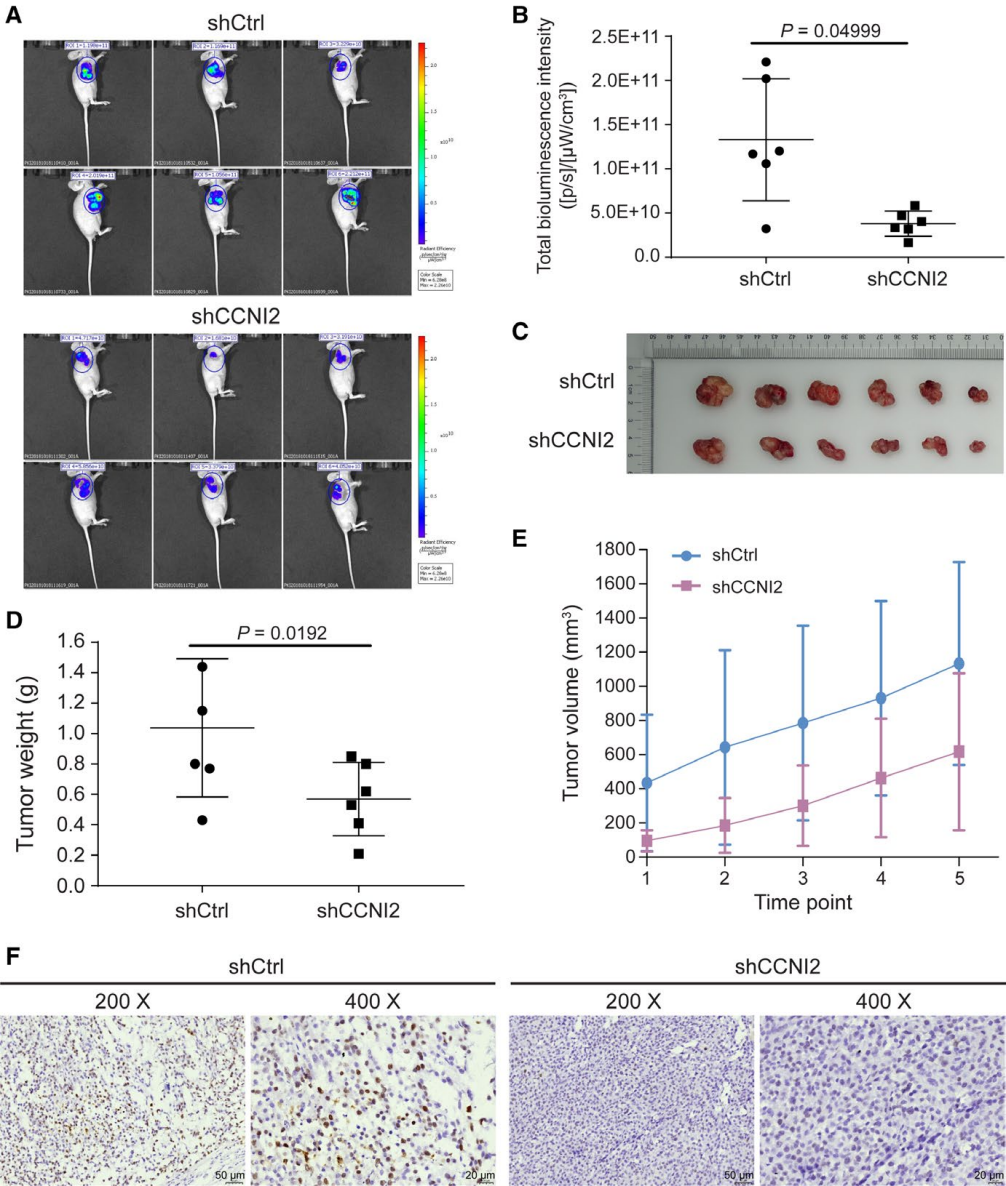
The influence of *CCNI2* knockdown on CRC *in vivo*. (A) *In vivo* imaging showed the growth of tumors in mice models. (B) The levels of bioluminescence intensity in shCCNI2 group were significantly decreased than that in control group. Statistical analysis was performed by unpaired *t*‐test (two‐tailed). (C) Representative images of tumors in subcutaneous xenograft mice models. (D) The changes of tumors weight in mice models. Statistical analysis was performed by unpaired *t*‐test (two‐tailed). (E) The changes of tumors volume in mice models during experimental session. (F) The expression level of Ki‐67 in tumor tissue of mice models was showed by immunohistochemical staining. The magnification was 200 times (scale bar =50 μm) and 400 times (scale bar =20 μm). ShCtrl: cells infected with scrambled control shRNA; shCCNI2: cells infected with *CCNI2*‐shRNA.

To further verify the effect of *CCNI2* on CRC *in vivo*, tumor‐bearing nude mouse models with or without *CCNI2* knockdown were constructed. On the 28^th^ day after subcutaneous injection, 15 mg/mL D‐Luciferin were injected into the abdominal cavity of mice. The *in vivo* image results were presented in Figure 4A, which showed the growth of tumors in nude mice models. Besides, the bioluminescence intensity in shCCNI2 group was significantly decreased than that in control group (Figure 4B) which suggested that downregulation of *CCNI2* expression suppressed CRC growth. After 28 days, all mice were sacrificed and solid tumors were collected (Figure 4C). The weight and volume of tumors were both smaller in shCCNI2 group compared to the control group (Figure 4D‐E). Furthermore, the immunohistochemical staining results revealed that Ki‐67 was downregulated in shCCNI2 group (Figure 4F), which was in consistence with the above results. Herein, all the results indicated that downregulation of *CCNI2* could impair the development of CRC *in vivo*.

## 4 DISCUSSION

Colorectal cancer (CRC) is the third most common malignant cancer in the world and the fourth leading cause of cancer‐associated death, but in the past decades there were relatively few advances in therapeutic strategies, especially for patients with advanced stages.^21^ The occurrence of cancer may be caused by mutations in pivotal oncogenes and tumor suppressor genes, leading to the dysregulation of cell proliferation and cell cycle and changes in protein expression.^22^ *TP53* was attested to inactivate by mutations or deletions in most human tumors and *POLR2A*, an important gene close to *TP53*, was always deleted together with *TP53*. Liu et al. presented that if the expression of *POLR2A* was inhibited by siRNA, the proliferation, survival, and tumorigenic potential of CRC cells with hemizygous *TP53* loss would be selectively inhibited in a p53‐independent manner.^23^ Despite considerable efforts, no effective therapeutic strategies of CRC have been developed.

Cyclins play a vital role in cell proliferation, cycle, and tumorigenesis in all eukaryotes.^22^ To investigate the effects of Cyclin I‐like (*CCNI2*), the homolog of Cyclin I (*CCNI*), on CRC, the immunohistochemical staining results of Paraffin‐embedded CRC tissues microarray indicated *CCNI2* had a high expression in CRC tissues. Furthermore, Kaplan‐Meier survival analysis indicated that patients with a high *CCNI2* expression level had poor prognosis. It suggested that *CCNI2* might be involved in the progression of CRC and had the potential as a prognostic marker. We also compared the expression of *CCNI2* in CRC cell lines with that in human normal colorectal mucosal cells before the study *in vitro*, which was found *CCNI2* had a higher expression in CRC cells. Whereafter, the interference sequences targeting *CCNI2* were designed to silence *CCNI2* expression in CRC cells. On this base, we tested the cell proliferation and colony formation ability of these cells. It was manifested that after the expression level of *CCNI2* decreased, the proliferation rate of CRC cells was significantly slowed down and the ability of cell colony was also inhibited. This was consistent with the finding of Liu et al. that knockdown of *CCNI2* by siRNA inhibited cell proliferation.^19^ In addition, results of cell cycle showed *CCNI2* silencing could increase G2 phase ratio significantly, suggesting that downregulation of *CCNI2* arrested cell cycle. Simultaneously, cell apoptosis results, detected by flow cytometry, revealed that *CCNI2* knockdown could promote CRC cell apoptosis. The similar results were manifested by Human apoptosis antibody array that *CCNI2* knockdown significantly increased the expression levels of BID, BIM, Caspase3, and DR6, and decreased IGF‐II expression level. In brief, *CCNI2* functioned on CRC cell apoptosis, but the further molecular mechanism was still unclear. Currently, it has been reported that *CCNI2* could be involved in cell cycle regulation *via* binding *CDK5*, thus, affecting cell proliferation and apoptosis.^19^ Studies have shown that *CDK5* modulated the ERK5 AP‐1 signaling axis to promote tumor multiplication, formation, and invasion of CRC.^24^ *CDK5*/*p35* complex, as a downstream gene of *IC53*, overexpression promoted CRC proliferation.^25^ However, further research was still needed.

During the investigation of mice xenograft model, fluorescence images analysis and solid tumor weight measure results manifested that tumor growth in mice was memorably inhibited after knockdown of *CCNI2*. Downregulation of Ki‐67 expression levels detected by immunohistochemical staining suggested that the proliferation of CRC cells in the mice model was inhibited by knockdown of *CCNI2*. Based on the results of *in vitro* and *in vivo* experiments, we found that *CCNI2* participated in the development of CRC by regulating the proliferation and apoptosis of CRC cells. As the homolog of *CCNI2*, *CCNI* was verified to be able to influence MAPK signaling pathway to regulate the expression of pro‐survival proteins Bcl‐2 and Bcl‐XL, which could prevent injury‐induced apoptosis.^26^ Other study has shown that the *CCNI2* downstream gene, *CDK5*, had an antiapoptotic effect by indirectly inhibiting peroxisome proliferator‐activated receptor g (PPARg).^27^

In conclusion, *CCNI2* was significantly upregulated in the CRC. The promoting effects of *CCNI2* on CRC were investigated and verified in this study, suggesting that *CCNI2* might be a potential diagnostic and prognostic indicator for CRC. However, the downstream molecules and specific regulatory mechanisms of *CCNI2* in CRC need to be further explored.

## CONFLICT OF INTEREST

The authors declare no conflicting of interests.

## AUTHOR'S CONTRIBUTION

D.M. Lai made a substantial contribution to the hypothesis and design of this study. Y.X. Tong implemented the experiments involved in this study. J.J. Bi, Y.H. Chen, and Y.D. Wu collected, organized, and analyzed experimental data. Q.W. Huang, H.J. Li, S. Zhang, and Z. Fu wrote and revised the paper. All of the authors reviewed the results, read and approved the final manuscript.

## Supporting information

Fig S1

Fig S2

Fig S3

Fig S4

Fig S5

Fig S6

## Data Availability

The data that support the findings of this study are available from Y.X. Tong, upon reasonable request.
