# Supplementary figures and images for: CCNI2 plays a promoting role in the progression of colorectal cancer
Source: Cancer Med. 2021 Feb 23;10(6):1913–24. doi: 10.1002/cam4.3504 (PMC7957193; doi:10.1002/cam4.3504)

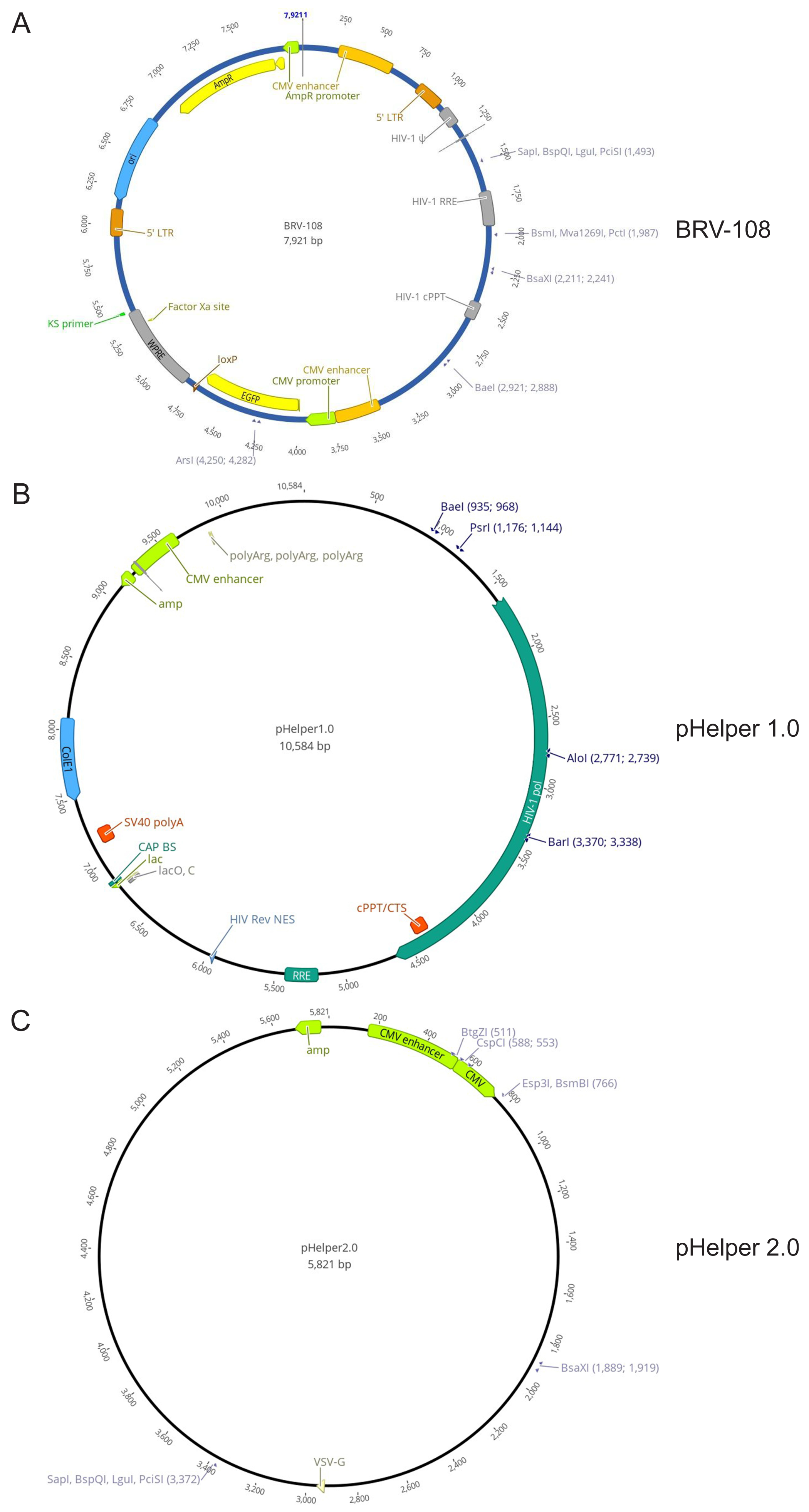

Supplement: Supplementary file 1 — Fig S1 [file CAM4-10-1913-s006.tif]

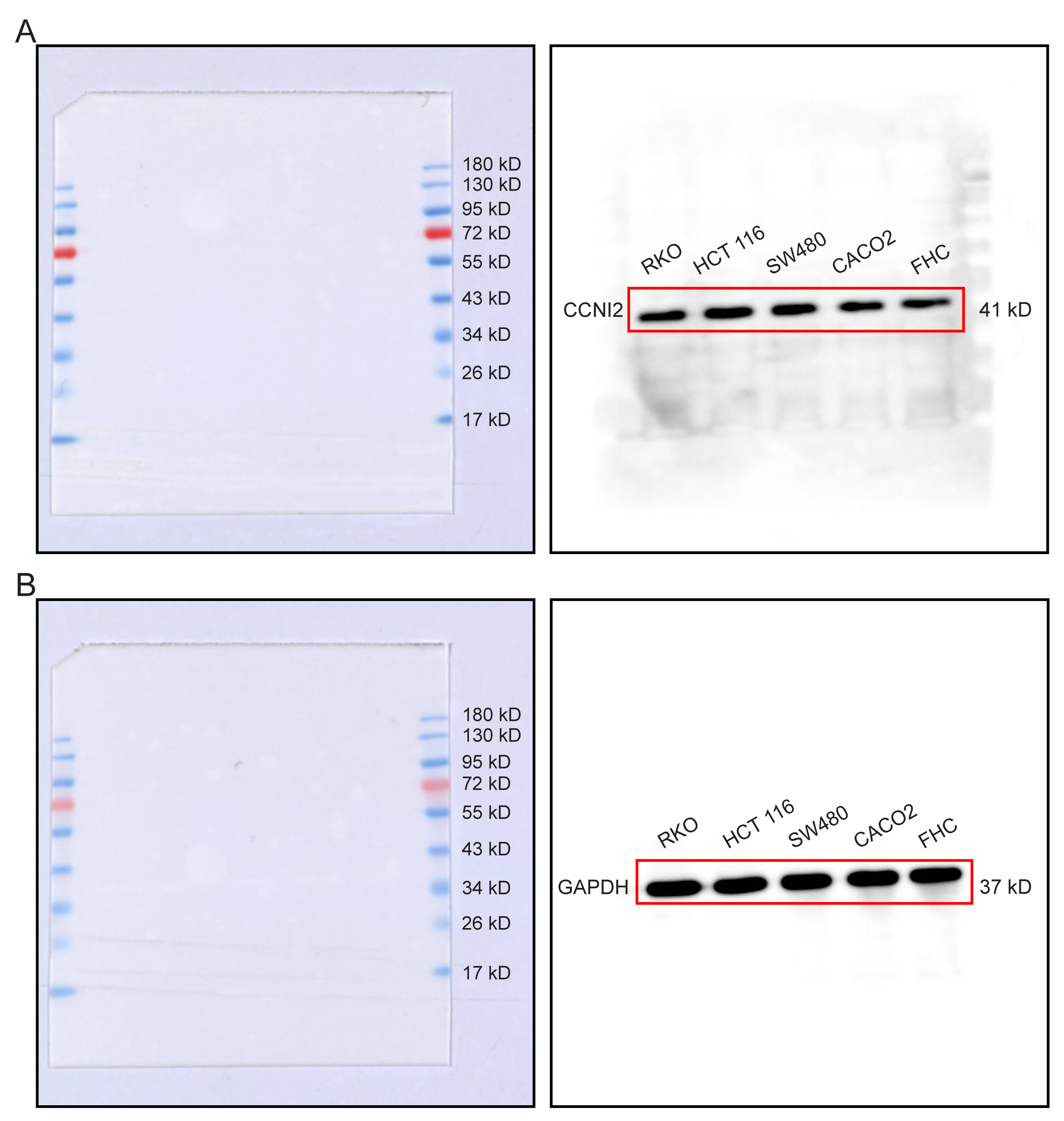

Supplement: Supplementary file 2 — Fig S2 [file CAM4-10-1913-s003.tif]

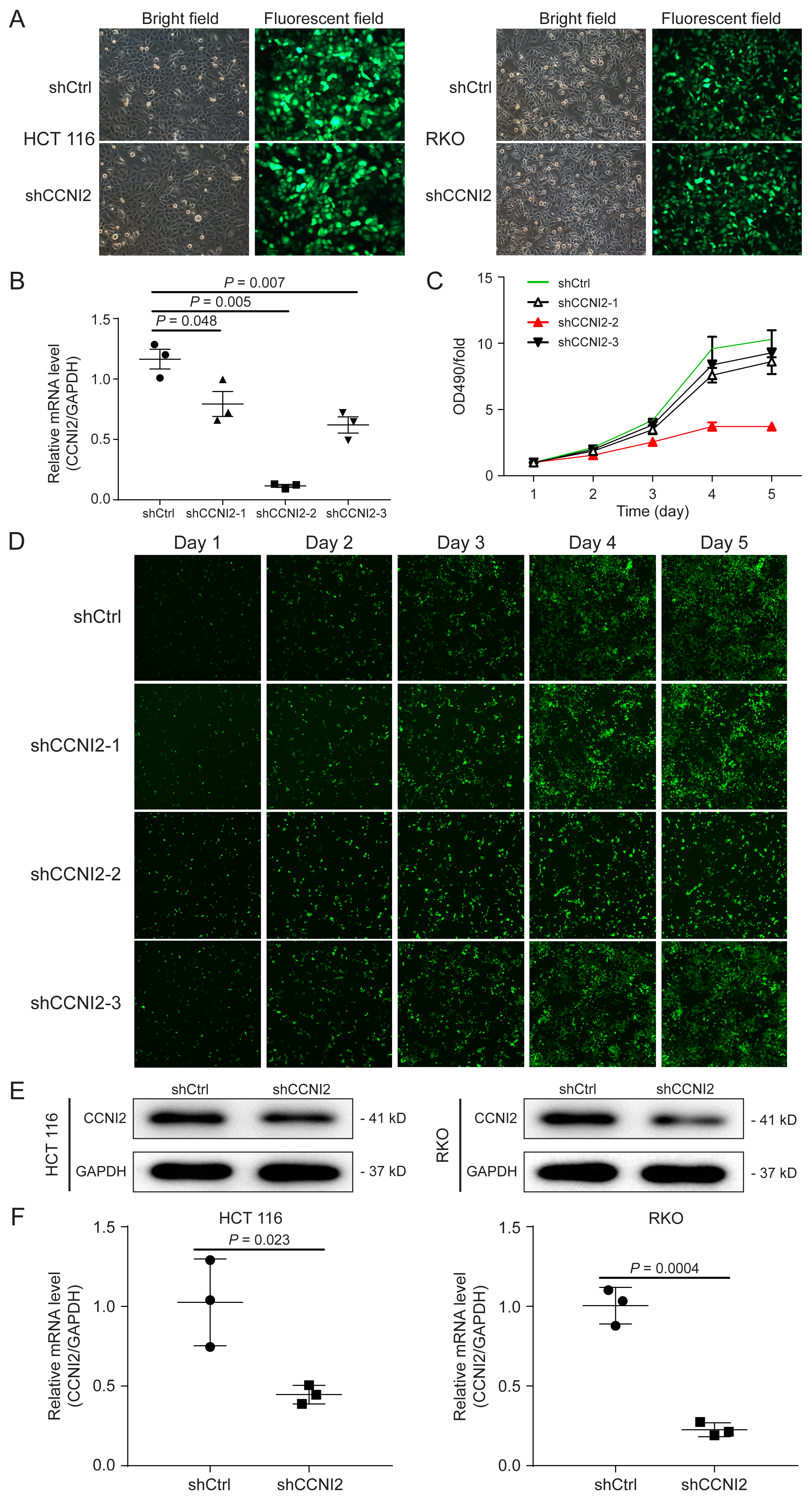

Supplement: Supplementary file 3 — Fig S3 [file CAM4-10-1913-s001.tif]

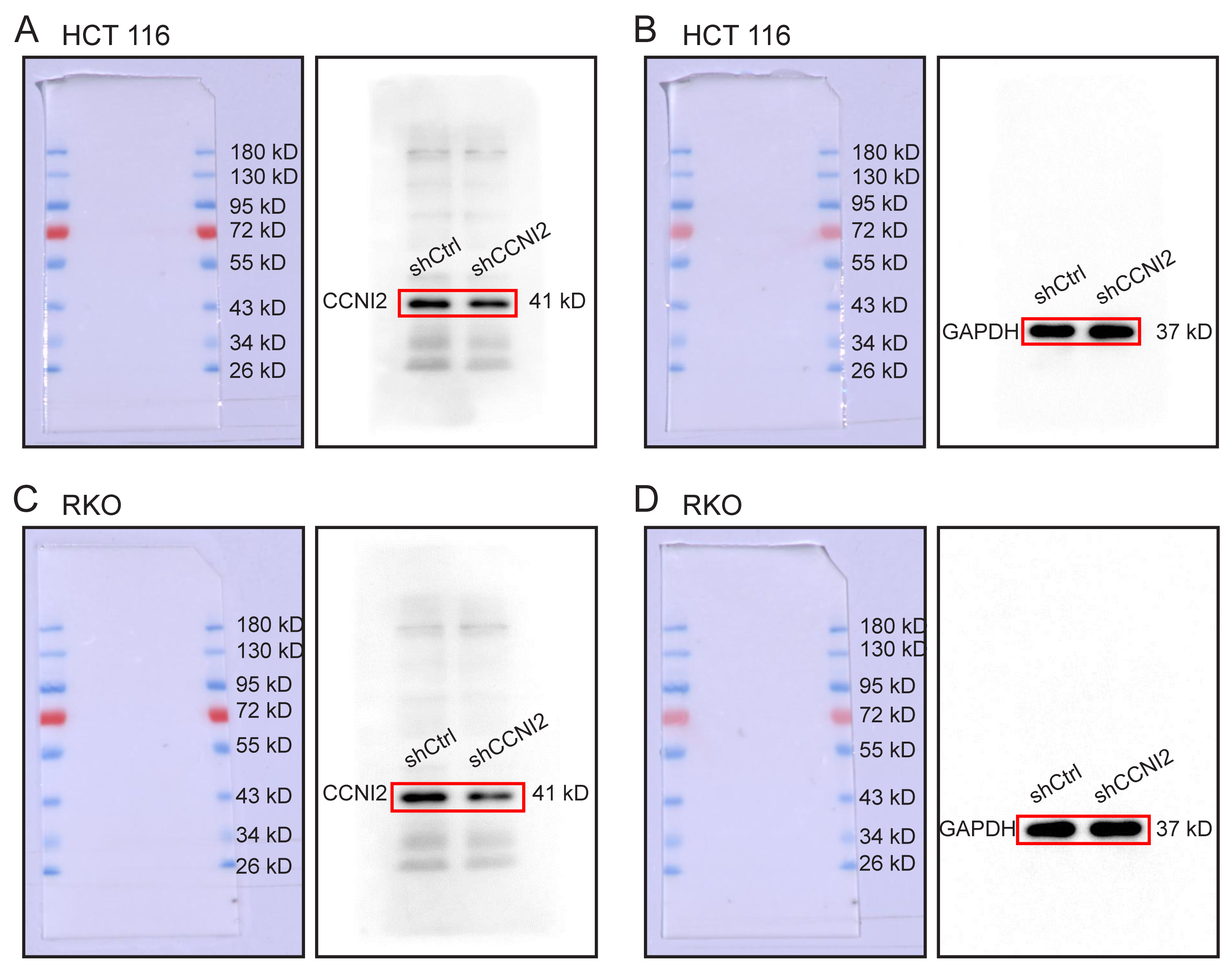

Supplement: Supplementary file 4 — Fig S4 [file CAM4-10-1913-s005.tif]

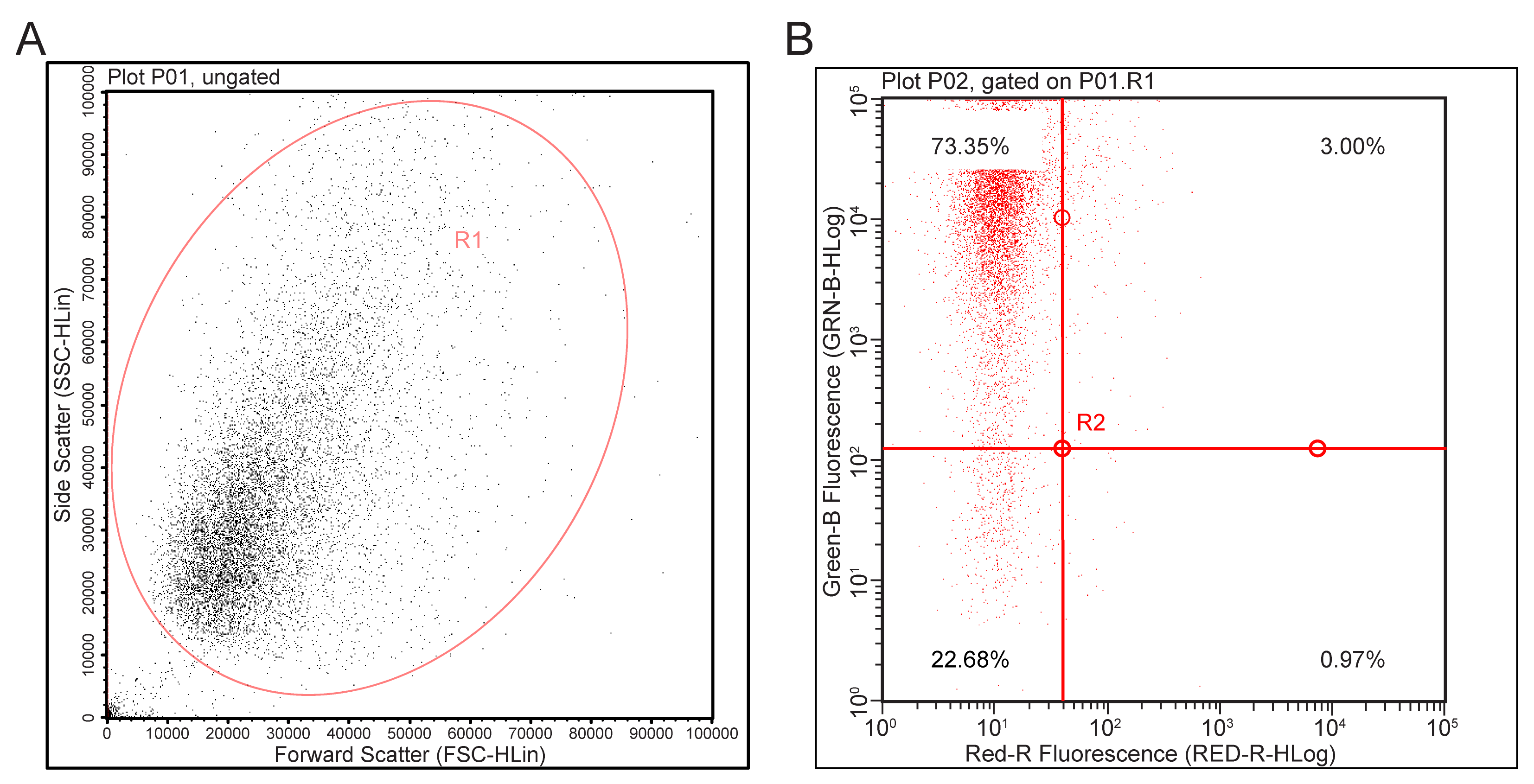

Supplement: Supplementary file 5 — Fig S5 [file CAM4-10-1913-s004.tif]

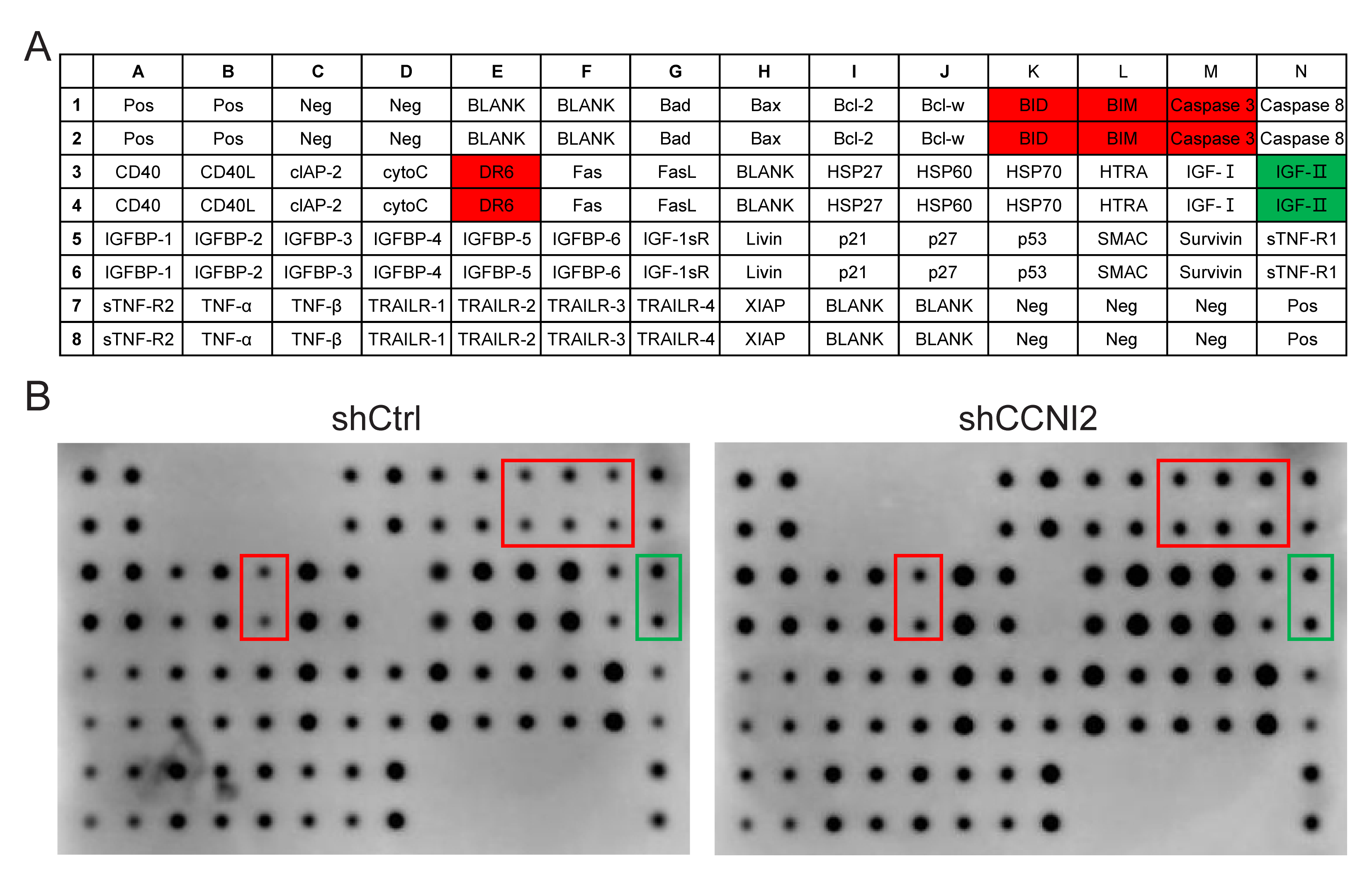

Supplement: Supplementary file 6 — Fig S6 [file CAM4-10-1913-s002.tif]
